# Heart-Protective Mechanical Ventilation in Postoperative Cardiosurgical Patients

**DOI:** 10.1155/2021/6617809

**Published:** 2021-03-23

**Authors:** Vadim Mazurok, Igor Kasherininov, Andrey Bautin, Olga Kulemina, Ryta Rzheutskaya

**Affiliations:** Almazov National Medical Research Centre, Akkuratova St, 2, Saint Petersburg 197341, Russia

## Abstract

**Background:**

This study compared the hemodynamic effects and gas exchange under several different ventilator settings—with regard to tidal volume, respiratory rate, and end-expiratory pressure—in patients after coronary artery bypass grafting (CABG).

**Methods:**

Prospective interventional cohort study with a controlled group in a single cardiosurgical ICU involving 119 patients following on-pump CABG surgery. During the 1st postoperative hour, the intervention group patients were ventilated with Vt 10 ml × kg^−1^, RR 14/min, PEEP 5 cmH_2_O (“conventional ventilation”). During the 2nd hour, RR was reduced to 8/min (“reduced RR ventilation”). At 3 hrs, Vt was decreased to 6 ml × kg^−1^, RR returned to 14/min, and PEEP increased to 10 cmH_2_O (“low Vt-high PEEP ventilation”).

**Results:**

Patients in the “low Vt-high PEEP” ventilation period showed significantly lower alveolar ventilation and thoraco-pulmonary compliance than during “reduced RR” ventilation. Mean airway pressure and Vds/Vt peaked during low Vt-high PEEP ventilation; however, driving pressure was lower. Vt decrease and PEEP increase did not lead to oxygenation improvement and worsened CO_2_ elimination. Hemodynamically, the study revealed significant cardiac output decrease during low Vt-high PEEP ventilation. In 23.2% of patients, catecholamine therapy was initiated.

**Conclusions:**

In postoperative cardiosurgical patients, MV with Vt 6 ml × kg^−1^ and PEEP 10 cm H_2_O is characterized by worsened oxygenation and elimination of CO_2_ and a less favorable hemodynamic profile than ventilation with Vt 10 ml × kg^−1^ and PEEP 5 cmH_2_O. *New and Noteworthy*. (i) Patients after CABG may be especially sensitive to low tidal volume and increased PEEP as it negatively affects hemodynamic profile by means of the right heart preload decrease and afterload increase. (ii) Mechanical ventilation settings aiming to minimize mean airway pressure reduce the negative effects of positive inspiratory pressure and are favorable for hemodynamics.

## 1. Introduction

Mechanical ventilation (MV) affects both respiratory and cardiovascular systems as a result of positive inspiratory pressure [1]. The “lung-protective strategy” characterized by the use of a relatively small tidal volume (Vt) of 6 ml × kg^−1^ and by variable adjustment of positive end-expiratory pressure (PEEP) is spreading widely across different categories of patients [2]. This strategy helps to defend the lungs from baro- and volumotrauma and to improve outcomes in patients with acute respiratory distress syndrome (ARDS) [3]. The lung-protective approach is also recommended to patients without gas exchange disorders and those who undergo surgery under anesthesia [4]. According to the lung-protective model, the main predictor of pulmonary complications is a driving pressure (ΔР: the difference between inspiratory plateau pressure and PEEP) of more than 15 cm H_2_O [5].

The results of the PReVENT trial conducted to determine whether a ventilation strategy using low Vt (4–6 ml × kg^−1^ of predicted body weight (PBW)) is superior to one with intermediate Vt (8–10 ml × kg^−1^ PBW) in critically ill patients without ARDS [6] did not demonstrate an advantage to a low Vt strategy. In addition, other concerns about low Vt persist, including the increase in sedation needs and the incidence of delirium in ICU [7], the increase in ICU-acquired weakness [8], and patient-ventilator asynchrony [9], and the risk of lung tissue collapse [10]. Therefore, the use of low Vt ventilation leads to increased sedation use due to ventilator dyssynchrony. This has been associated with an increased incidence of delirium with benzodiazepine use. Today, it is uncertain whether ventilation with lower Vt (≤6 ml × kg^−1^) should be used routinely in all ICU patients, and lung-protective strategy is not recommended in guidelines for perioperative patients without ARDS.

Due to the interdependence of heart-lung physiology—in which an increased intrathoracic pressure has a depressing effect on cardiac output (CO), affecting right heart performance especially [11–13]—one of the most serious problems in postcardiac surgery care is the setting of proper MV parameters for patients with compromised respiratory and hemodynamic profiles, and particularly in those having decreased myocardial contractility.

Positive expiratory pressure, set to prevent alveolar collapse, leads to right atrium preload decrease and right ventricle (RV) afterload increase [1, 11]. However, due to quite sophisticated and often unpredictable heart-lung interaction [14], high PEEP values during the recruitment maneuver may, in contrast, improve RV performance [15, 16].

As patients undergoing coronary artery bypass grafting (CABG) often exhibit multiple risk factors contributing to potential respiratory complications [17–19] and hemodynamic instability [5], the setting of optimal MV parameters for such patients is a clinical challenge. Therefore, the value of the lung-protective ventilation strategy in postoperative CABG patients having neither ARDS nor severe hemodynamic disorders needs to be determined.

This prospective study compared the hemodynamic effects and gas exchange under several different ventilator settings in postcardiac surgery patients.

## 2. Patients and Methods

### 2.1. Patients

This prospective study was approved by the local Ethics Committee of Almazov National Medical Research Centre and included 119 on-pump CABG patients' data during the years 2016-2017. We included ICU patients who had undergone CABG surgery. All patients signed informed consent prior to surgery.

The following exclusion criteria were defined:

- Acute myocardial infarction
- Symptomatic congestive heart failure
- Baseline left ventricle ejection fraction (LVEF) < 40%
- Baseline PaO_2_/FiO_2_ < 300 mmHg
- Baseline pulmonary hypertension (mean PAP ≥25 mmHg)
- Complex surgery: CABG with valve replacement
- Age >80 years
- Postoperative doses of inotropic drugs above moderate and/or increased doses of vasopressors (norepinephrine >0.5 *μ*g × kg^−1^ × min^−1^, phenylephrine >0.7 *μ*g × kg^−1^ × min^−1^)
- Use of mechanical hemodynamic support devices
- Significant arrhythmias (AV-blockade, atrial fibrillation, high-grade ventricular extrasystolia, and ventricular tachycardia)

### 2.2. Surgical Period

Intraoperatively, before and after cardiopulmonary bypass (CPB), mechanical ventilation was performed by anesthesia ventilator «Datex Ohmeda ADU Care station» (GE Healthcare, USA) using volume controlled mode, with PEEP 5 cm H_2_O, Vt 8 ml × kg^−1^, FiO_2_ 0.4–0.6, SpO_2_ 97–99%. In compliance with the local protocol, during the period of CPB, the mechanical ventilation was stopped. The extracorporeal circulation was performed via standard cannulation (ascending aorta, right atrium) by CPB machine «Stockert S V» (Sorin Group, Germany) with varying membrane oxygenators («Dideco», «Maquet», «Terumo») under moderate hypothermia (34°C) and total heparinization (Activated Clotting Time >480 sec). Mean perfusion pressure was maintained aiming 70 mm Hg, CPB flow 2.4–2.5 l/min × m^2^, PaO_2_ 150–250 mm Hg, PaCO_2_ 33–38 mm Hg. Heart protection was achieved with intermittent antegrade and retrograde isothermic blood cardioplegia using KCl solution.

Infusion therapy during the intra- and postoperative periods was standard and consisted predominantly of balanced crystalloids. The average positive fluid balance by the end of surgery was 1-2 l.

Following surgery, patients were transferred to ICU.

#### 2.2.1. Protocol

In the initial phase, 95 patients were included (the intervention group). After calculating and analyzing the initial data, 24 extra patients with the same baseline criteria were added to the control group to confirm the obtained results.

**Figure 1:**
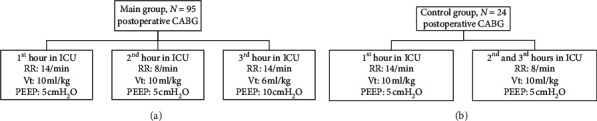
MV settings in patients.

MV in the postoperative period was carried out using SIMV mode by ICU ventilator MV200 (Triton Electronic Systems, Russia). MV settings in patients of both the initial and control groups are presented in Figure 1.

The MV parameters during the 1^st^ and 2^nd^ study hours were quite traditional for both groups: in the 1^st^ hour, Vt was 10 ml × kg^−1^, PEEP 5 cm H_2_O, and RR 14/min, representing the “conventional ventilation” period. During the second hour, for reducing mean airway pressure (*P*_mean_), RR was decreased to 8/min: the “reduced RR ventilation” period. During the 3^rd^ postsurgical hour, MV parameters in the intervention group were changed in accordance with lung-protective strategy: Vt decreased to 6 ml × kg^−1^, PEEP increased to 10 cmH_2_O, RR returned to 14/min—“low Vt-high PEEP ventilation.”

MV settings in the control group patients did not change between the 2^nd^ and 3^rd^ hours. Patients in this prolonged “reduced RR ventilation” maintained the lowest *P*_mean_, calculated by ventilators as the ratio of inspiratory pressure (*P*_insp_) time to expiratory pressure time.

During the study period, all patients had residual sedation after high opiate anesthesia; some needed additional sedation (propofol 0.3–0.9 mg × kg^−1^ × hour^−1^).

#### 2.2.2. Measurements

At the end of each hour, the hemodynamic parameters (CO, stroke volume, pulmonary artery pressure, systemic and pulmonary vascular resistance, mean arterial (MAP), and central venous (CVP) pressure) and respiratory parameters (*P*_insp_, PEEP, airway resistance (R), compliance (C), alveolar (Va) and dead space (Vd) ventilation, and ΔР), as well as arterial blood gases were evaluated.

Prepulmonary thermodilution was used to measure the hemodynamic parameters. Respiratory parameters were measured via respirators, in particular by means of volumetric capnometry.

The hemodynamic parameters of 50 patients out of 95 (52.6%) from the intervention group, who did not require inotropes or vasopressors throughout all study periods, were evaluated to outline the “native” hemodynamic profile without “noise” ensuing from catecholamine use.

Catecholamines (dobutamine) were introduced in the case of a persistent (>10 minutes) MAP decrease below 65 mmHg. Inotropic therapy was initiated when the cardiac index (CI) decreased below 2.4 l × min^−1^ × m^−2^. In a case of CI ≥ 2.4 l × min^−1^ × m^−2^, an infusion of vasopressors (Norepinephrine) was administered.

Before awakening, patients were weaned in accordance with the local protocol: MV in SIMV mode (Vt 8-9 ml × kg^−1^, RR 11–14/min, PEEP 5 cm H_2_O), FiO_2_ 0.4–0.5. After 30 minutes of effective CPAP ventilation, with FiO_2_ 0.3–0.4, patients were extubated.

### 2.3. Statistical Analysis

It was carried out with the Microsoft Office Excel (Microsoft, USA) and Statistica 7.0 programs (Statsoft Inc., USA). Student parametric criteria were used for normal distribution and the Wilcoxon test for abnormal distribution. A *P* value lower than 0.05 was considered significant. Data are presented as median values with 25th and 75th percentiles.

## 3. Results

The data on the respiratory parameters of 95 screened patients from the interventional group during the study are presented in Tables 1 and 2.

As shown in Table 1, during the low Vt-high PEEP ventilation period (the 3^rd^ study hour) the interventional group patients showed statistically significant lower alveolar ventilation and compliance than during reduced RR ventilation (the 2^nd^ hour). By contrast, the Vds/Vt ratio and *P*_mean_ increased during low Vt-high PEEP ventilation; however, Δ*Р* was lower here than during reduced RR ventilation.

Table 2 details the parameters of acid-base balance, oxygenation, and CO_2_ elimination in patients from the interventional group. These data show that Vt decrease and PEEP increase during the 3^rd^ hour did not cause an improvement in oxygenation: PaO_2_/FiO_2_ appeared to be less than during the 2^nd^ hour. In addition, the low Vt-high PEEP period was characterized by the worst CO_2_ elimination, as well as by the development of acidosis between the 2^nd^ and 3^rd^ hrs.

Twenty from 95 patients (21.1%) required catecholamine therapy during the “conventional ventilation period” (the 1^st^ ICU hour). In the reduced RR ventilation phase, three more patients required catecholamines, raising the total requirement in inotropes to 24.2%. During low Vt-high PEEP, 45 patients (47.4%) received inotropes and/or vasopressors. Thus, the transition from MV with Vt 10 ml × kg^−1^ and PEEP 5 cm H_2_O to Vt 6 ml × kg^−1^ and PEEP 10 cm H_2_O led to initiation of catecholamine therapy in 22 patients (23.2%).

Hemodynamic profile changes in 50 of the main-group patients not requiring catecholamines are presented in Table 3.

According to the subgroup analysis seen in Table 3, central venous pressures, mean pulmonary artery pressures, and pulmonary artery wedge pressures, as well as pulmonary vascular resistance, appeared to be highest during low Vt-high PEEP ventilation. At the same time, in patients not requiring catecholamines, cardiac output and stroke volume decreased significantly.

In contrast to those exposed to low Vt-high PEEP ventilation, the 24 patients of the control group with unchanged MV settings between the 2^nd^ and 3^rd^ hours demonstrated no significant changes in either respiratory mechanics, gas exchange, or hemodynamic profile at the 3^rd^ stage (Tables 4–6). No patient from this group required catecholamine therapy initiation.

All included patients were extubated at 6–9 hours after surgery.

## 4. Discussion

The obtained data reveal that postoperative CABG patients without baseline severe respiratory and hemodynamic disorders demonstrated the worst cardiovascular and oxygenation parameters during the “low Vt-high PEEP ventilation” period. In contrast, the optimal cardiopulmonary parameters were obtained during the “reduced RR ventilation” period, when *P*_mean_ was the lowest.

The minimal PaO_2_/FiO_2_ level that occurred during the “conventional ventilation” period (the first hour after surgery) can be caused by atelectatic changes induced by numerous factors of on-pump cardiac surgery [17, 19]. Nevertheless, hemodynamic and respiratory parameters were statistically better during the reduced RR ventilation period, probably as a cumulative result of the cardiorespiratory function in the case of the lowest positive inspiratory and mean airway pressure.

As other factors (infusion rate, volume status, estimated blood loss, and sedation level) follow the same pattern, the obtained data are obviously the product of a change in *P*_mean_ resulting from altered MV settings. Vt decrease and PEEP increase during low Vt-high PEEP ventilation were accompanied by a significant *P*_mean_ and Vds/Vt increase. These changes led to a significant etCO_2_ and PaCO_2_ increase, caused hypercapnia in some cases, and resulted in higher incidence and severity of mixed acidosis. All these symptoms are well-known effects of ventilation with small tidal volume.

The changes in Δ*Р*, as well as their influence on gas exchange, deserve special attention. Thus, in the recent literature, the increase in Δ*P* is considered as a predictor of adverse outcomes in patients with respiratory disorders [5]. In our study, Vt decrease and PEEP increase logically led to Δ*P* decrease—accompanied, however, by a deterioration of gas exchange. These findings are explicable as well: our patients had no restrictive respiratory failure, so lung injury due to relatively high Δ*P* was elusive.

The hemodynamic changes do seem to be the most important entity. In the absence of other obvious causes, it can be assumed that hemodynamic parameters are largely determined by the level of PEEP and increased *P*_mean_. Within heart-lung interaction, higher *P*_mean_ (mean intrathoracic pressure) correlates to an impaired venous return. Despite the formally increased preload parameters for the right and left chambers (CVP and PAWP), during the low Vt-high PEEP ventilation phase, there are significant decreases in CO, stroke volume, and mean arterial pressure. These results are consistent with the well-known fact that intrathoracic pressure increase produces a false increase in the atrium load, which does not permit considering CVP as a true index of RV preload [1], or PAWP of LV preload.

Thus, our data make an input to continuing debates regarding the preload indicators in mechanically PEEP-ventilated patients in modern literature [1]. In particular, both Vt increase [20] and PEEP increase [12] are mentioned as causes of the discrepancy between CVP and PAWP readings and the real values of the ventricles' preload. In our study, higher PEEP, unlike increased Vt, appeared to act as a leading factor of circulatory disorders.

An increased PVR, reflecting the RV afterload increase, should be considered as another significant adverse factor for cardiosurgical patients. A possible explanation for PVR rise is the influence of increased *P*_mean_ on the elastic pulmonary vessels with increasing RV afterload. At the same time, a number of studies devoted to lung recruitment and the use of increased PEEP describe either the opposite effect [21] or the absence of such PEEP effect on the RV afterload [13].

However, it is less clear which mechanical ventilation settings are optimal for patients with healthy lungs. Simonis with coworkers in a recent review state that the effect of PEEP seems opposite; higher PEEP is beneficial in ARDS patients, but not in patients without ARDS. In patients with healthy lungs, PEEP could cause overdistension, thereby increasing Δ*Р* and compromising the hemodynamic system that could lead to harmful effects [22]. While a low to moderate level of PEEP may prevent lung injury through the reduction of atelectasis, higher PEEP is undeniably associated with an increased risk of intraoperative hypotension that frequently requires administration of vasoactive drugs [23].

Similar results were received in a recent randomised clinical trial (RCT) of patients scheduled for one-lung ventilation during oesophagectomy; patients in the protective ventilation group (Vt 6 ml kg^−1^ PBW) had a greater need for vasopressors and also developed hypercapnia more frequently than patients in the conventional ventilation group (Vt 10 ml kg^−1^ PBW) [24]. Among adult patients undergoing major surgery, intraoperative ventilation with low tidal volume (6 ml kg^−1^ PBW) compared with conventional tidal volume (10 ml·kg^−1^ PBW), with PEEP applied equally between groups, did not significantly reduce pulmonary complications within the first 7 postoperative days [25].

Taken all together, this suggests that low Vt and increased PEEP should be used only in accordance with strict indications—namely, in the case of restrictive respiratory failure. In other words, lung-protective strategy cannot be automatically extrapolated onto other patient groups, in particular to stable cardiosurgical patients.

Lastly, judgment regarding “heart protection” is impossible without additional diagnostic instruments: heart ultrasound, troponine, B-type natriuretic peptide, etc. Therefore, the term “heart-protective ventilation” in our context means rather the paramount importance of caution in the selection of MV settings for patients who have undergone cardiac surgery.

Limitations of the study include a small sample size and a relatively short period of observations. Though the time of mechanical ventilation in the groups was the same, we did not assess other clinical endpoints of ICU/hospital stay and mortality. The study design aimed to assess the effect of several different interventions, lower tidal volume, higher PEEP, and reduced *P*_mean_, rather than trying to assess only one variable and keep the others controlled. Therefore, it is quite difficult to assess the relative effect of the different parameters. The optimal cardiopulmonary parameters were obtained during the “reduced RR ventilation” period. While this may be useful over a one-hour period, it is unclear whether a prolonged period of low *P*_mean_ in CABG patients might lead to a higher rate of atelectasis/basal lung collapse, hence delaying recovery. Finally, cardiorespiratory profile and acid-base balance in cardiosurgical patients during the 1^st^ and 3^rd^ hours after surgery may not be the same. Thus, to counteract this discrepancy, we introduced the control group.

## 5. Conclusions

Due to the physiologic effects, lung-protective strategy, created for ARDS treatment, should not be routinely used in post-CABG patients. Even in patients without severe respiratory and hemodynamic problems at baseline, MV with Vt 6 ml × kg^−1^ and PEEP 10 cm H_2_O exhibits a less favorable hemodynamic profile, as compared to the strategy with Vt 10 ml × kg^−1^ and PEEP 5 cm H_2_O. A significant CO, CI, SV, and MAP decrease and RV afterload increase and preload decrease can be regarded as a moderate RV dysfunction often requiring catecholamine therapy initiation.

## Figures and Tables

**Table 1:** Changes in respiratory parameters in patients from the interventional group at three hours of the study, median (25^th^; 75^th^ percentiles), *n* = 95.

Data	Hours in ICU			
	#1	#2	#3	*P*
MV (l × min^−1^)	11.2 (10.1; 12.6)	6.4 (5.8; 7.2)	6.7 (6.1; 7.6)	*P*_3−1_, *P*_3−2_, *P*_2−1_ <0.001

Va (l × min^−1^)	9.5 (8.8; 10.7)	5.4 (5.0; 6.1)	5.2 (4.7; 5.9)	*P*_3−1_, *P*_3−2_, *P*_2−1_ <0.001

*P*_mean_ (cmH_2_O)	10.0 (10.0; 11.0)	8.0 (8.0; 9.0)	13.0 (13.0; 14.0)	*P*_3−1_, *P*_3−2_, *P*_2−1_ <0.001

*P*_insp_ (cmH_2_O)	21.0 (20.0; 23.0)	21.0 (19.0; 22.0)	21.0 (19.0; 23.0)	*P*_3−1_ = 0.5 *P*_3−2_ = 0.06 *P*_2−1_ <0.001

R (cmH_2_O × l^−1^ × sec^−1^)	7.9 (6.5; 8.5)	7.4 (6.4; 8.6)	7.0 (6.0; 8.0)	*P*_3−1_, *P*_3−2_ <0.001 *P*_2−1_ = 0.007

С (ml × cmH_2_O^−1^)	54.1 (46.7; 62.1)	56.7 (49.0; 62.4)	54.1 (48.2; 59.8)	*P*_3−1_ = 0.2 *P*_3−2_ <0.001 *P*_2−1_ = 0.006

Vds/Vte (%)	14.0 (12.0; 15.0)	14.0 (13.0; 16.0)	21.0 (18.0; 24.0)	*P*_3−1_, *P*_3−2_ <0.001 *P*_2−1_ = 0008

Δ*P* (cmH_2_O)	16.0 (15.0; 18.0)	16.0 (14.0; 17.0)	11.0 (9.0; 13.0)	*P*_3−1_, *P*_3−2_, *P*_2−1_ <0.001

MV: minute ventilation; Va: alveolar ventilation; *P*_mean_: mean airway pressure; *P*_insp_: inspiratory pressure; R: resistance; C: compliance; Vds/Vte: dead space/tidal volume ratio; Δ*Р*: driving pressure; *P*: Wilcoxon test.

**Table 2:** Changes in oxygenation, elimination of CO_2_, and acid-base balance of arterial blood in patients from the interventional group, median (25^th^; 75^th^ percentile), *n* = 95.

Data	Hours in ICU			
	#1	#2	#3	*P*
PaO_2_/FiO_2_ (mmHg)	237.0 (184.0; 333.3)	324.0 (274.0; 372.0)	292.0 (232.0; 346.0)	*P*_3−1_, *P*_3−2_, *P*_2−1_ <0.001

PaCO_2_ (mmHg)	30.0 (28.0; 34.0)	36.0 (33.2; 40.0)	39.0 (35.0; 43.0)	*P*_3−1_, *P*_3−2_, *P*_2−1_ <0.001

etCO_2_ (mmHg)	27.0 (24.0; 29.0)	34.0 (32.0; 37.0)	38.0 (34.0; 40.0)	*P*_3−1_, *P*_3−2_, *P*_2−1_ <0.001

Arterial рН	7.45 (7.40; 7.48)	7.38 (7.34; 7.41)	7.34 (7.32; 7.39)	*P*_3−1_, *P*_3−2_, *P*_2−1_ <0.001

РаСО_2_ >45 mmHg rate	0	4.2%	13.7%	*P*_3−1_ <0.001 *P*_3−2_ = 0.023 *P*_2−1_ = 0.044

Arterial рН <7.35 rate	2.1%	26.3%	52.6%	*P*_3−1_, *P*_3−2_, *P*_2−1_ <0.001

Arterial ВЕ	−1.6 (−3.2; −0.1)	−3.1 (−4.3; −1.5)	−3.4 (−4.7; −1.9)	*P*_3−1_ <0.001 *P*_3−2_ = 0.2 *P*_2−1_ <0.001

Arterial НСО_3_^−^	21.0 (20.0; 23.0)	21.0 (20.0; 22.0)	21.0 (20.0; 22.0)	*P*_3−1_ = 0.4 *P*_3−2_ = 0.4 *P*_2−1_ = 0.1

PaO_2_/FiO_2_: respiratory index; PaCO_2_: partial pressure of carbon dioxide in arterial blood; etCO_2_: expired carbon dioxide; *P*: the Wilcoxon test.

**Table 3:** Changes in hemodynamic profile in patients from the interventional group not requiring catecholamines for three hours of study, median (25^th^; 75^th^ percentile), *n* = 50.

Data	Hours in ICU			
	#1	#2	#3	*P*
HR (bpm)	80 (69; 86)	77 (71; 85)	76 (72; 86)	*P*_3−1_ = 0.7 p_3-2_ = 0.5 *P*_2−1_ = 0.97

MAP (mmHg)	85 (79; 97)	83.5 (76; 91)	73 (69; 82)	*P*_3−1,3−2_ <0.001 *P*_2−1_ = 0.2

PAP mean (mmHg)	16 (13; 18)	17 (14; 19)	19 (16; 22)	*P*_3−1,3−2_ <0.001 *P*_2−1_ = 0.06

CVP (mmHg)	6 (5; 8)	6 (4; 7)	8 (6; 9)	*P*_3−1,3−2_ <0.001 *P*_2−1_ = 0.7

PAWP (mmHg)	7 (5; 9)	8 (6; 9)	10 (7; 11)	*P*_3−1,3−2_ <0.001 *P*_2−1_ = 0.1

CO (l × min^−1^)	5.05 (4.30; 5.90)	5.20 (4.40; 5.90)	4.65 (4.30; 5.30)	*P*_3−1_ = 0.001 *P*_3−2_ <0.001 *P*_2−1_ = 0.21

CI (l × min^−1^ × m2^−1^)	2.6 (2.4; 2.9)	2.7 (2.4; 3.0)	2.5 (2.2; 2.7)	*P*_3−1_ = 0.002 *P*_3−2_ <0.001 *P*_2−1_ = 0.2

SV (ml)	66 (54; 80)	65 (54; 78)	60 (54; 67)	*P*_3−1_ = 0.002 *P*_3−2_ <0.001 *P*_2−1_ = 0.9

SI (ml × m2^−1^)	34 (29; 39)	35 (29; 40)	31 (29; 35)	*P*_3−1_ = 0.009 *P*_3−2_ <0.001 *P*_2−1_ = 0.5

SVR (dyn × sec^−1^ × cm^−5^)	1254 (1077; 1509)	1142 (1071; 1426)	1088 (963; 1341)	*P*_3−1_ <0.001 *P*_3−2_ = 0.001 *P*_2−1_ = 0.06

PVR (dyn × sec^−1^ × cm^−5^)	127.5 (111; 163)	134.5 (109; 157)	152.5 (136; 183)	*P*_3−1,3−2_ <0.001 *P*_2−1_ = 0.98

HR: heart rate; MAP: mean arterial pressure; PAP: pulmonary artery pressure; CVP: central venous pressure; PAWP: pulmonary artery wedge pressure; CO: cardiac output; CI: cardiac index; SV: stroke volume; SI: stroke index; SVR: systemic vascular resistance; PVR: pulmonary vascular resistance; *P*: Wilcoxon test.

**Table 4:** Changes in respiratory mechanics data in patients from the control group at three hours of the study, median (25^th^; 75^th^ percentile), *n* = 24.

Data	Hours in ICU			
	#1	#2	#3	*P*
MV (l × min^−1^)	10.5 (10.1; 12.0)	6.2 (5.8; 7.0)	6.2 (5.8; 7.0)	*P*_3−1,2−1_ <0.001 *P*_3−2_ >0.5

Va (l × min^−1^)	9.2 (8.6; 10.1)	5.2 (5.0; 5.8)	5.2 (4.9; 5.8)	*P*_3−1,2−1_ <0.001 *P*_3−2_ = 0.54

*P*_mean_ (cmH_2_O)	10.5 (10.0; 11.0)	9.0 (8.0; 10.0)	9.0 (8.0; 10.0)	*P*_3−1,2−1_ <0.001 *P*_3−2_ = 0.54

*P*_insp_ (cmH_2_O)	22.0 (20.0; 23.0)	21.0 (20.0; 22.0)	21.5 (20.0; 22.0)	*P*_3−1_ = 0.062 *P*_3−2_ = 0.65 *P*_2−1_ = 0.002

R (cmH_2_O × l^−1^ × sec^−1^)	7.7 (6.9; 8.6)	7.4 (6.8; 8.3)	7.9 (7.1; 8.4)	*P*_3−1_ = 0.95 *P*_3−2_ = 0.032 *P*_2−1_ = 0.086

С (ml × cmH_2_O^−1^)	53.5 (49.3; 56.8)	52.7 (49.9; 56.8)	53.4 (50.8; 57.2)	*P*_3−1_ = 0.27 *P*_3−2_ = 0.11 *P*_2−1_ = 0.92

Vds/Vte (%)	15.0 (14.0; 17; 0)	15.0 (13.0; 16.0)	15.0 (13.0; 16.0)	*P*_3−1_ = 0.36 *P*_3−2_ = 1 *P*_2−1_ = 0.36

Δ*P* (cmH_2_O)	14.1 (13.2; 17.0)	14.3 (12.9; 17.6)	14.0 (12.7; 17.6)	*P*_3−1_ = 0.3 *P*_3−2_ = 0.06 *P*_2−1_ = 0.98

MV: minute ventilation; Va: alveolar ventilation; *P*_mean_: mean airway pressure; *P*_insp_: inspiratory pressure; R: resistance; C: compliance; Vds/Vte: dead space/tidal volume ratio; Δ*Р*: driving pressure; *P*: Wilcoxon test.

**Table 5:** Changes in oxygenation, elimination of CO_2_, and acid-base balance of arterial blood in patients from the control group, median (25^th^; 75^th^ percentile), *n* = 24.

Data	Hours in ICU			
	#1	#2	#3	*P*
PaO_2_/FiO_2_ (mmHg)	245.0 (211.0; 298.5)	284.0 (242.0; 326.0)	284.0 (260.5; 348.5)	*P*_3−1,2−1_ <0.001, *P*_3−2_ = 0.03
PaCO_2_ (mmHg)	29.9 (28.5; 32.4)	35.9 (34.3; 37.8)	36.6 (34.4; 38.8)	*P*_3−1,2−1_ <0.001, *P*_3−2_ = 0.32
etCO_2_ (mmHg)	27.0 (25.0; 29.0)	33.0 (31.8; 34.3)	33.5 (32.0; 35.0)	*P*_3−1,2−1_ <0.001, *P*_3−2_ = 0.15
Arterial рН	7.45 (7.40; 7.48)	7.41 (7.37; 7.43)	7.40 (7.37; 7.43)	*P*_3−1,2−1_ <0.001, *P*_3−2_ = 0.15
РаСО_2_ >45 mmHg (rate)	4.2%	16.7%	20.8%	*P*_3−1_ = 0.081, *P*_3−2_ = 0.712, *P*_2−1_ = 0.157
Arterial рН <7.35 (rate)	0	4.2%	0	*P*_3−1_ = 1, *P*_3−2,2−1_ = 0.313
Arterial ВЕ	−0.85 (−2.0; 0.8)	−1.4 (−2.6; −0.8)	−2.2 (−3.7; −1.1)	*P*_3−1_ <0.001, *P*_3−2_ = 0.01, *P*_2−1_ = 0.02
Arterial HCO_3_^−^	22.4 (21.4; 23.6)	21.9 (21.3; 23.2)	21.8 (20.9; 22.8)	*P*_3−1_ = 0.003, *P*_3−2_ = 0.03, *P*_2−1_ = 0.2

PaO_2_/FiO_2_: respiratory index; PaCO_2_: partial pressure of carbon dioxide in arterial blood; etCO_2_: expired carbon dioxide; P: Wilcoxon test.

**Table 6:** Changes in hemodynamic profile in patients from the control group, median (25^th^; 75^th^ percentile), *n* = 24.

Data	Hours in ICU			
	#1	#2	#3	*P*
HR (bpm)	78.0 (69.8; 84.0)	76.5 (72.0; 82.3)	76.0 (70.8; 82.3)	*P*_3−1_ = 0.57 *P*_3−2_ = 0.85 *P*_2−1_ = 0.54

MAP (mmHg)	82.5 (77.5; 89.0)	82.0 (75.5; 86.0)	80.0 (75.3; 86.3)	*P*_3−1_ = 0.035 *P*_3−2_ = 0.31 *P*_2−1_ = 0.034

PAP mean (mmHg)	19.0 (15.0; 20.3)	18.5 (15.0; 19.3)	17.5 (15.0; 20.0)	*P*_3−1_ = 0.31 *P*_3−2_ = 0.72 *P*_2−1_ = 0.2

CVP (mmHg)	7.0 (5.0; 8.3)	6.5 (6.0; 8.0)	7.0 (5.8; 8.0)	*P*_3−1_ = 0.61 *P*_3−2_ = 0.42 *P*_2−1_ = 0.17

PAWP (mmHg)	8.5 (5.0; 10.0)	8.0 (5.0; 8.3)	7.5 (5.8; 8.3)	*P*_3−1_ = 0.19 *P*_3−2_ = 0.79 *P*_2−1_ = 0.035

CO (l × min^−1^)	5.2 (4.3; 5.8)	5.3 (4.7; 5.9)	5.6 (4.6; 6.2)	*P*_3−1_ = 0.034 *P*_3−2_ = 0.74 *P*_2−1_ = 0.19

CI (l × min^−1^ × m2^−1^)	2.75 (2.4; 3.0)	2.75 (2.4; 3.0)	2.85 (2.5; 3.1)	*P*_3−1_ = 0.034 *P*_3−2_ = 0.77 *P*_2−1_ = 0.17

SV (ml)	67.0 (59.8; 74.3)	69.5 (60.0; 78.0)	70.5 (61.0; 82.5)	*P*_3−1_ = 0.01 *P*_3−2_ = 0.84 *P*_2−1_ = 0.13

SI (ml × m2^−1^)	34.5 (31.6; 37.3)	35.8 (33.3; 38.8)	35.8 (33.8; 41.0)	*P*_3−1_ = 0.008 *P*_3−2_ = 0.81 *P*_2−1_ = 0.12

SVR (dyn × sec^−1^ × cm^−5^)	1177 (1020; 1346)	1120 (951; 1240)	1070 (980; 1179)	*P*_3−1_ <0.001 *P*_3−2_ = 0.22 *P*_2−1_ = 0.007

PVR (dyn × sec^−1^ × cm^−5^)	167.0 (131.3; 190.5)	175.0 (115.5; 188.5)	163.0 (114.0; 184.0)	*P*_3−1_ = 0.26 *P*_3−2_ = 0.41 *P*_2−1_ = 0.42

HR: heart rate; MAP: mean arterial pressure; PAP: pulmonary artery pressure; CVP: central venous pressure; PAWP: pulmonary artery wedge pressure; CO: cardiac output; CI: cardiac index; SV: stroke volume; SI: stroke index; SVR: systemic vascular resistance; PVR: pulmonary vascular resistance; P: Wilcoxon test.

## Data Availability

The data used to support the findings of this study are available on request to the corresponding author.

## References

[1] Pinsky M. R. (2014). My paper 20 years later: effect of positive end-expiratory pressure on right ventricular function in humans. *Intensive Care Medicine*.

[2] Fuller B. M., Ferguson I. T., Mohr N. M. (2017). Lung-protective ventilation initiated in the emergency department (LOV-ED): a quasi-experimental, before-after trial. *Annals of Emergency Medicine*.

[3] Slutsky A. S., Ranieri V. M. (2000). Mechanical ventilation: lessons from the ARDSNet trial. *Respiratory Research*.

[4] Neto A. S., Cardoso S. O., Manetta J. A. (2012). Association between use of lung-protective ventilation with lower tidal volumes and clinical outcomes among patients without acute respiratory distress syndrome. *JAMA*.

[5] Amato M. B. P., Meade M. O., Slutsky A. S. (2015). Driving pressure and survival in the acute respiratory distress syndrome. *New England Journal of Medicine*.

[6] Simonis F. D., Serpa Neto A., Binnekade J. M. (2018). Effect of a low vs intermediate tidal volume strategy on ventilator-free days in intensive care unit patients without ARDS. A randomized clinical trial. *JAMA*.

[7] Seymour C. W., Pandharipande P. P., Koestner T. (2012). Diurnal sedative changes during intensive care: impact on liberation from mechanical ventilation and delirium. *Critical Care Medicine*.

[8] Lipshutz A. K. M., Gropper M. A. (2013). Acquired neuromuscular weakness and early mobilization in the intensive care unit. *Anesthesiology*.

[9] Kallet R. H., Campbell A. R., Dicker R. A., Katz J. A., Mackersie R. C. (2006). Effects of tidal volume on work of breathing during lung-protective ventilation in patients with acute lung injury and acute respiratory distress syndrome. *Critical Care Medicine*.

[10] Kallet R. H., Siobal M. S., Alonso J. A., Warnecke E. L., Katz J. A., Marks J. D. (2001). Lung collapse during low tidal volume ventilation in acute respiratory distress syndrome. *Respiratory Care*.

[11] Repessé X., Charron C., Vieillard-Baron A. (2016). Acute respiratory distress syndrome: the heart side of the moon. *Current Opinion in Critical Care*.

[12] Cherpanath T. G. V., Lagrand W. K., Binnekade J. M., Schneider A. J., Schultz M. J., Groeneveld J. A. B. (2016). Impact of positive end-expiratory pressure on thermodilution-derived right ventricular parameters in mechanically ventilated critically ill patients. *Journal of Cardiothoracic and Vascular Anesthesia*.

[13] Reis Miranda D., Gommers D., Struijs A. (2004). The open lung concept: effects on right ventricular afterload after cardiac surgery. *British Journal of Anaesthesia*.

[14] Grübler M. R., Wigger O., Berger D., Blöchlinger S. (2017). Basic concepts of heart-lung interactions during mechanical ventilation. *Swiss Medical Weekly*.

[15] Longo S., Siri J., Acosta C. (2016). Lung recruitment improves right ventricular performance after cardiopulmonary bypass. *European Journal of Anaesthesiology*.

[16] Schulman D. S., Biondi J. W., Matthay R. A., Barash P. G., Zaret B. L., Soufer R. (1988). Effect of positive end-expiratory pressure on right ventricular performance: importance of baseline right ventricular function. *The American Journal of Medicine*.

[17] Barbosa R. A. G., Carmona M. J. C. (2002). Evaluation of pulmonary function in patients undergoing cardiac surgery with cardiopulmonary bypass. *Revista Brasileira de Anestesiologia*.

[18] Arom K. V., Emery R. W., Petersen R. J., Schwartz M. (1995). Cost-effectiveness and predictors of early extubation. *The Annals of Thoracic Surgery*.

[19] Bautin A. E., Kasherininov I. Yu., Laletin D. A. (2016). Prevalence and structure of acute respiratory failure in the early postoperative period of cardiosurgical interventions. *Vestnik Intensivnoy Terapii*.

[20] Lansdorp B., Hofhuizen C., van Lavieren M. (2014). Mechanical ventilation-induced intrathoracic pressure distribution and heart-lung interactions. *Critical Care Medicine*.

[21] Tusman G., Bohm S. H., Suarez Sipmann F. (2006). Alveolar recruitment decreases pulmonary vascular resistance after cardiopulmonary bypass (Abstract). *Anesthesiology*.

[22] Simonis F. D., Juffermans N. P., Schultz M. J. (2021). Mechanical ventilation of the healthy lungs: lessons learned from recent trials. *Current Opinion in Critical Care*.

[23] Hol L., Nijbroek S. G. L. H., Schultz M. J. (2020). Perioperative lung protection: clinical implications. *Anesthesia & Analgesia*.

[24] van der Woude M. C., Bormans L., van der Horst R. P. (2020). Pulmonary levels of biomarkers for inflammation and lung injury in protective versus conventional one-lung ventilation for oesophagectomy: a randomised clinical trial. *European Journal of Anaesthesiology*.

[25] Karalapillai D., Weinberg L., Peyton P. (2020). Effect of intraoperative low tidal volume vs conventional tidal volume on postoperative pulmonary complications in patients undergoing major surgery: a randomized clinical trial. *JAMA*.

